# Mesenteric dermoid cyst in a child

**Published:** 2011-11-21

**Authors:** Damien Punguyire, Kenneth Victor Iserson

**Affiliations:** 1 Kintampo Municipal Hospital, Kintampo, Ghana; 2 Department of Emergency Medicine The University of Arizona, USA

**Keywords:** Cyst, mesentery, dermoid, pediatrics, tumor

## Abstract

If a pediatric abdominal mass is not organomegaly or colonic stool, narrowing the diagnostic possibilities may be difficult, especially in resource-poor areas where ancillary tests and treatment options may be limited. A 2-year-old girl was brought to the rural Kintampo Municipal Hospital in Ghana with a freely moveable, non-tender abdominal mass. A huge mesenteric dermoid cyst was surgically removed. Mesenteric cysts are rare intra-abdominal lesions, most commonly occurring in children <10 years old. Making a preoperative diagnosis is difficult. Dermoid cysts (mature cystic teratoma) rarely occur in the mesentery. Poverty, family circumstances and the rural location led to general physicians doing surgery. As in this case, due to economic, social and transportation issues common throughout Africa, children with abdominal masses may need at least initial surgery in hospitals without dedicated pediatric surgery or even a trained surgeon.

## Background

Parents often bring their infants and children to clinicians after noticing an abdominal mass. Most of these are either organomegaly - a number that is probably much higher in malaria endemic areas - or colonic stool. Those that cannot be easily identified must be evaluated further, often with surgical exploration. Ninety percent of those requiring surgery are retroperitoneal, with about half originating in the urinary tract [1]. Abdominal masses in young children pose a diagnostic and therapeutic challenge in resource-poor regions.

Narrowing the diagnostic possibilities requires a careful medical history and physical examination. In resource-poor areas, all desired ancillary testing may be difficult or impossible to obtain. Treatment options may also be restricted due to poverty, geographic location, the parents’ personal responsibilities (e.g., other young children at home), and the professional staff's capabilities.

We describe such a case in a rural district/municipal West African hospital with limited imaging capacity and a general physician who also acts as a surgeon. It illustrates the diagnostic/therapeutic process that led to an unusual diagnosis and a good patient outcome.

## Patient and case report

A 2-year-old girl was brought to the Kintampo Municipal (District) Hospital in rural Ghana because the child's mother noticed a mass in the abdomen. The mother related that she had noticed it for some time, but wasn't sure for how long. She stated that the child had no changes in her eating or defecation pattern, no bleeding or bruising, fever, night sweats, behavioral changes or any other pertinent medical history.

**Figure 1:**
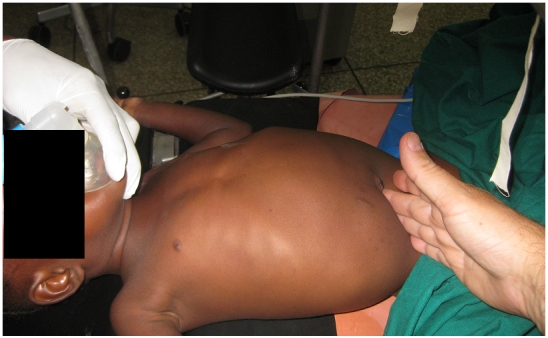
Clinician's hand indicating the inferior tip of the abdominal mass in a child with mesenteric dermoid cyst

On physical exam, the 10 Kg child had a normal physical exam other than a 10 x 5 cm mass overlying the right colon (Figure 1). It appeared to be mobile and non-tender. There was no evidence of an underlying infectious process, bowel obstruction, organomegaly, mechanical difficulties with breathing, lymphadenopathy, or compromised venous return from the mass. The mass caused no venous return or intestinal motility. The liver could be felt as a discrete organ.

Her urinalysis was normal and a complete blood count demonstrated a Hgb 10.1 g/dL, WBC 7.2 x 10^9^/L, and a normal differential. An ultrasound demonstrated a non-specific, probably cystic mass in the abdomen without any other identifiable pathology. An upright radiograph showed displaced bowel. No other laboratory tests or imaging modalities were available at this hospital.

Clinicians suggested that the child be taken to another facility, but the mother's finances, family situation, and reluctance to go elsewhere precluded that option. When it became clear that the mother would not take the child to the closest hospital with better diagnostic and treatment facilities, the decision was made to operate. The general physician, who also does surgery, performed the procedure.

**Figure 2:**
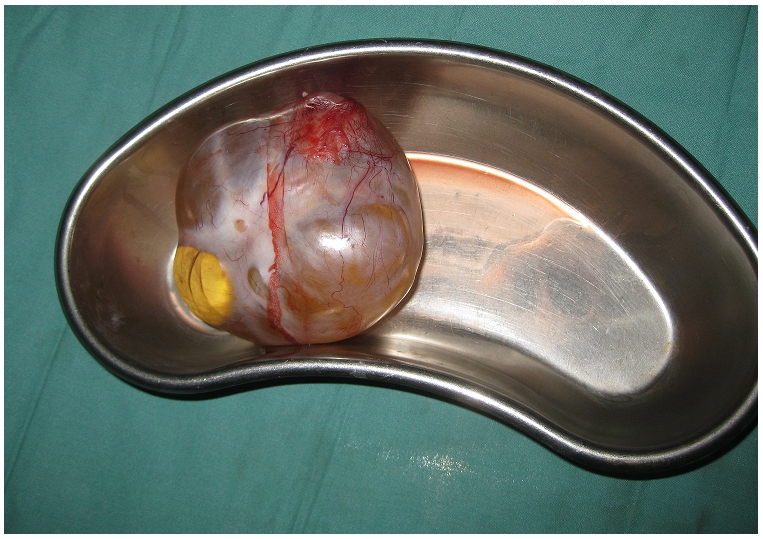
Mesenteric dermoid cyst after removal

Given the prevalence of parasitosis in the population and the child's rather benign presentation, the preoperative diagnosis was a probable parasitic cyst. The child was brought to the theater where, under general anesthesia, a midline laparotomy was performed. A large, smooth, glistening mass was immediately apparent occupying the right side and around 1/3 of the total abdomen. It was isolated from the iliac mesentery and removed intact (Figure 2). It weighed 1Kg. The child had an uneventful recovery. The specimen was sent to a pathologist at another hospital.

The pathology report showed a “huge cystic mass measuring about 10 cm in diameter and roughly spherical in shape containing serous fluid. Cut section reveals copious serous fluid.” The final diagnosis was a mesenteric dermoid cyst without evidence of malignancy.

## Discussion

### Differential Diagnosis

The differential diagnosis for an abdominal mass in a 2-year-old child (Table 1) often can be significantly shortened using the history, physical examination, and available imaging. Laboratory tests, if available, can be of some assistance. In most cases, treatment is, at least partially, surgical. In this case, the child did not demonstrate any systemic signs of illness, debility or weight loss. One could account for the low hemoglobin as being at, or even above the norm for 2-year-old children in the local population. In part, this was due to widespread malaria, but poor nutrition also plays a role. These findings tend to eliminate the most malignant tumors and aggressive infections. Normal oral intake eliminates the various types of bowel obstruction.

**Table 1:** Differential diagnosis of abdominal mass in a small child

Gastrointestinal	
Appendiceal abscess	Intussusception
Burkitt's lymphoma	Malrotation
Fecal mass	Meckel's diverticulum
Giant lymphoid hyperplasia	Pyloric stenosis
Hirschsprung disease	Rhadomyosarcoma
Inflammatory bowel disease	Teratoma (stomach/liver)
Intestinal duplication	Volvulus
	
**Genital**	
Hydrometrocolpos	Ovarian teratoma
Ovarian cyst	Ovarian torsion
	
**Hepatobiliary**	
Angiosarcoma	Hepatocellular carcinoma
Cholecystitis	Hepatomegaly
Choledochal cyst	Hydrops of the gallbladder
Cystadenocarcinoma	Other hepatobiliary tumor
Embryonal sarcoma	Pancreatic pseudocyst
Focal nodular hyperplasia	Splenomegaly
Hepatoblastoma (<3 years old)	
	
**Renal**	
Horseshoe kidney	Renal cyst
Hydronephrosis	Wilms tumor
Polycystic kidney	
	
**Non-renal retroperitoneal**	
Lymphangioma	Neuroblastoma/ganglioneuroma/ganglioma
Lymphoma	Pancreatic pseudocyst
Megaureter	Teratoma
	
**Other**	
Abscess	Omental torsion
Anterior meningomyelocele	Toxic megacolon
Desmoid tumor	Tubereculosis
Hydatid cyst	Umbilical hernia
Inguinal hernia/hydrocele	Urachal Cyst
Omenatal/mesenteric cyst	Yersinia pseudotuberculosis mesenteric lymphadenitis

On physical examination, the child's mass was large, freely mobile, apparently non-tender, and not associated with other organomegaly. That would seem to eliminate confusing enlarged organs of any etiology, displaced organs (e.g., horseshoe kidney), or fixed masses (e.g., pancreatic pseudocyst). Its mobility also significantly lessens the probability that the mass was of retroperitoneal origin.

The limited available imaging (and expertise at interpretation) suggested that the mass was a single cystic structure. Combined with the other available information, this led to the preoperative presumptive diagnosis of a hydatid (Echinococcal) cyst not associated with the liver. Key elements in this presumed diagnosis were that parasitism is rampant in the local population and that patients with hydatid cysts can be asymptomatic, despite very large masses. Admittedly, hydatid cysts are usually parenchymal (i.e., liver or lung), so this finding would be unusual. However, the practical importance of considering this diagnosis prior to surgery was that we were intent on keeping the cyst intact, since intraperitoneal rupture might precipitate anaphylactic shock.

As pathological evaluation subsequently showed, the cyst was actually a giant mesenteric dermoid cyst, perhaps an even rarer diagnosis.

### Mesenteric Dermoid Cysts

Mesenteric cysts are rare intra-abdominal lesions, occurring most frequently (∼75%) in white patients >10 years old; these lesions are rare in black patients [2]. They can occur anywhere in the mesentery, from the duodenum to the rectum, and may extend into the retroperitoneum. Mesenteric cysts have an incidence of 1/105,000 hospital admissions [2]. Nearly two-thirds of pediatric mesenteric cysts occur in males (62.5%), and 75% of children with mesenteric cysts are less than 5 years old [3]. Mesenteric cysts have a 3% malignancy rate [4]. A correct preoperative diagnosis of mesenteric cyst is rarely made at least without a modern ultrasound or CT scan evaluation [4].

Florentine anatomist Benevieni first described a mesenteric cyst in 1507, while performing an autopsy on an 8-year-old boy [4]. In 1880, Tillaux was the first to describe successfully resecting a mesenteric cyst [5]. In 2000, DePerrot, et al., suggested a classification for mesenteric cysts based on histopathological features: (a) cysts of lymphatic origin (simple lymphatic cyst and lymphangioma); (b) cysts of mesothelial origin (simple mesothelial cyst, benign cystic mesothelioma, and malignant cystic mesothelioma); (c) cysts of enteric origin (enteric cyst and enteric duplication cyst); (d) cysts of urogenital origin; (e) mature cystic teratoma (dermoid cysts), and (f) pseudocysts (infectious and traumatic cysts) [6].

Dermoid cysts (mature cystic teratoma) contain developmentally mature skin with its accompanying structures: hair follicles, sweat glands, hair, and often bits of other tissue. Since it contains mature tissue, they are almost always benign. This presentation of a dermoid cyst is very unusual. Dermoid cysts rarely present as mesenteric cysts [7].

### Diagnosis

Mesenteric cysts have varied presentations, ranging from an asymptomatic mass to an acute abdomen [8]. A lack of characteristic clinical features and radiological signs for mesenteric cysts make preoperative diagnosis difficult [9]. Mesenteric cysts usually present as incidental findings or with nonspecific and chronic symptoms. The most common presenting signs and symptoms are abdominal pain, a palpable abdominal mass or distention, nausea and vomiting, constipation, and diarrhea [8, 10].

Upright abdominal radiographs generally demonstrate a homogenous mass displacing bowel loops. Ultrasound examinations can generally diagnose a cystic mass, but may be problematic in further defining the pathology, especially if used by clinicians less experienced in ultrasound diagnosis [10]. Computerized tomography (CT scan) of the abdomen with intravenous contrast is the preoperative diagnostic method of choice, when available. If oral contrast is used, it may demonstrate the relationship of the cyst and intestines, allowing a conclusive diagnosis of a mesenteric cyst [8].

### Treatment in a Resource-Poor Setting

The treatment of choice for mesenteric cysts is resection. In the resource-poor settings typical of much of the developing world, a child's best option may often be to have surgery, despite a paucity of diagnostic tools and limited surgical capabilities. In this case, surgical intervention was appropriate, given that the diagnosis was unlikely to be a malignant tumor and might have been a low-grade infection requiring removal (as well as subsequent medical treatment). That the outcome was a diagnostic rarity was surprising.

The literature suggests, however, that, on occasion, the surgery for mesenteric cysts may be more complex, sometimes requiring bowel resection, marsupialization of the cyst, or even a subtotal gastrectomy [8, 10]. Yet it also indicates that “early identification of this rare cyst of unknown etiology may lead to the removal of a potentially malignant lesion, as well as reducing the unnecessary discomfort of intermittent abdominal pain” [9].

One further note is in order concerning the lack of resources. While obtaining pathology reports on surgical specimens is routine in many areas of the world, it was a complex procedure in this setting. The mother had to agree to pay for the analysis. Then the sample had to be sent to a hospital several hours away. Retrieving the pathologist's report was also an ordeal, since they initially couldn't locate it. That, however, is all part of working in rural sub-Saharan Africa.

## Conclusion

Mesenteric dermoid cysts are uncommon. In rare cases, these cysts can become malignant. They can present asymptomatically or with signs and symptoms suggestive of other intra-abdominal pathology. Preoperative diagnosis can be difficult, but can be made with expert ultrasonography or contrast CT scanning. Neither ultrasound experts nor CT scanning is available in most of sub-Saharan Africa, and especially not in rural areas. Post-operative (surgical pathology) diagnosis may be even more difficult, given the costs and logistics involved. Optimal treatment, both for definitive diagnosis and treatment, is intact removal, whenever possible. As in this case, due to economic, social and transportation issues that are common throughout Africa, children with abdominal masses may need at least initial surgery in hospitals without dedicated pediatric surgery or even a trained surgeon.
